# Loss of Sight and Enhanced Hearing: A Neural Picture

**DOI:** 10.1371/journal.pbio.0030048

**Published:** 2005-01-25

Stevie Wonder and Ray Charles are often cited as evidence that blindness confers superior musical ability. Wonder lost his sight after an incubator-related oxygen overdose during infancy; Charles lost his as a boy to glaucoma. It's impossible to know whether sight would have compromised their success, but many gifted musicians, from Jose Feliciano to Rahsaan Roland Kirk, lost their sight at an early age.

A number of human studies show that blind persons perform nonvisual tasks better than those with sight. Neuroimaging studies of blind persons performing nonvisual tasks, including hearing, show activity in brain areas normally associated with vision. But much remains to be learned about the nature and extent of this phenomenon: how these “visual areas” are used, the mechanisms that generate individual differences (not all blind persons can localize sounds better than the sighted, for example), and the neural processes that underlie it.

The task of localizing sound—which requires integrating information available to one ear only (monaural sounds available, for example, when one ear is plugged) or information derived from comparing sounds binaurally—is particularly suited to investigating the neural remapping that seems to follow vision loss. In a previous study, Franco Lepore and colleagues showed that people who lost their sight at an early age could localize sound, particularly from monaural cues, better than those who could see. These findings suggested that areas of the brain normally dedicated to processing visual stimuli (the visual cortex, located at the back of the brain in the occipital lobe) might play a role in processing sound in these individuals. In a new report, Lepore and colleagues use functional imaging studies to investigate the functional relationship between neural activity and enhanced hearing abilities in the blind, and find a strong correlation between superior sound localization skills and increased activity in the brain's visual center.

The authors hypothesized that if visual cortex recruitment bolstered auditory function in some individuals, then visual cortex activity would correlate with individual differences in performance, and the degree of activity should predict such differences. Nineteen people—seven sighted and twelve who lost their sight at an early age—were placed in an echo-free chamber and asked to indicate where a sound was coming from, using either one (monaural) or both (binaural) ears. The participants then performed the same tasks within a positron emission tomography (PET) machine, which measures brain activity through changes in cerebral blood flow (CBF).

Five of the blind participants could accurately localize sounds monaurally; most of the sighted could not. (All 19 participants had no trouble localizing binaural sounds.) Only the blind individuals with superior localization skills showed increased CBF in the visual cortex while performing monaural localization tasks. Interestingly, during binaural localization, the sighted participants showed decreased CBF in visual cortical areas. This decrease comports with previous studies showing that engaging one brain center—say, the temporal lobe, which processes sound—inhibits activation of others—such as the occipital lobe, which processes visual cues. These inhibitions appear to be absent in blind persons, though it's not clear why. It could be that blind persons don't need such inhibitions, the authors speculate, or maybe unrestricted access to the visual center serves to compensate for vision loss by boosting nonvisual senses.

Whether the enhanced auditory performance reported here simply reflects increased efficiency of auditory processing or indicates “supranormal” powers, Lepore and colleagues argue that their results show that the visual cortex is “specifically recruited to process subtle monaural cues more effectively.” It will be interesting to learn whether blind persons can recruit visual centers for other auditory tasks or to help them navigate the world without sight. Such studies would be vital for tailoring sensory support to suit individual needs and maybe even suggest ways to facilitate the neural cross talk that enhances auditory performance. But don't expect such innovations to recreate the likes of Rahsaan Kirk or Ray Charles anytime soon.

